# Cerebrospinal fluid endocannabinoid levels in Gilles de la Tourette syndrome

**DOI:** 10.1038/s41386-020-0671-6

**Published:** 2020-04-09

**Authors:** Kirsten R. Müller-Vahl, Laura Bindila, Beat Lutz, Frank Musshoff, Thomas Skripuletz, Charlotte Baumgaertel, Kurt-Wolfram Sühs

**Affiliations:** 1 Clinic of Psychiatry, Socialpsychiatry and Psychotherapy, Hannover Medical School, Hannover, Germany; 2 Institute of Physiological Chemistry, University Medical Center of the JGU, Mainz, Germany; 3 Forensic Toxicological Centre Munich, Munich, Germany; 4 Department of Neurology, Hannover Medical School, Hannover, Germany

**Keywords:** ADHD, Diagnostic markers

## Abstract

Gilles de la Tourette syndrome (TS) is a complex neurodevelopmental disorder characterized by the presence of motor and vocal tics as well as psychiatric comorbidities such as attention-deficit/hyperactivity disorder (ADHD), obsessive-compulsive disorder (OCD), depression, and anxiety. The underlying cause of the disease is still unknown, but several lines of evidence suggest a paramount role of the dopaminergic system. Based on the clinical observation that cannabis-based medicine including cannabis and delta-9-tetrahydrocannabinol (THC, dronabinol) may improve TS, alternatively, an involvement of the endocannabinoid system (ECS) has been suggested. In this study we measured cerebrospinal fluid (CSF) levels of the two most important endocannabinoids “N”-arachidonoylethanolamine (AEA, anandamide) and 2-arachidonoylglycerol (2-AG), the endocannabinoid-like molecule palmitoyl ethanolamide (PEA), and the lipid arachidonic acid (AA) in a sample of adult patients with TS (*n* = 20) compared with controls (*n* = 19) using liquid-liquid lipid extraction and simultaneous quantification by liquid chromatography multiple reaction monitoring (LC/MRM). CSF levels of AEA (*p* = 0.0018), 2-AG (*p* = 0.0003), PEA (*p* = 0.02), and AA (*p* < 0.0001) were significantly increased in TS compared with controls. Levels of 2-AG correlated with the severity of comorbid ADHD (*p* < 0.01). This is the first study, demonstrating alterations in the ECS suggesting an involvement of this system in the pathophysiology of TS. It can be speculated that elevated endocannabinoid levels either represent secondary changes in order to compensate for alterations in other neurotransmitter systems such as the dopaminergic system, are simply an epiphenomenon or, alternatively, represent the primary cause of TS.

## Introduction

Gilles de la Tourette syndrome (TS) is a neurodevelopmental disorder characterized by motor and vocal tics and different psychiatric comorbidities including attention-deficit/hyperactivity disorder (ADHD), obsessive-compulsive disorder (OCD), depression and anxiety. The underlying cause is still unknown, but most evidence supports a dopaminergic dysfunction. Since many patients are dissatisfied with established therapies, they seek for alternative treatments including cannabis-based medications. From open uncontrolled studies [1–7] and two small randomized controlled trials [8, 9], it is suggested that delta-9-tetrahydrocannabinol (THC, dronabinol), the most psychoactive ingredient of cannabis, but also cannabis and the cannabis extract nabiximols are effective in the treatment of tics and psychiatric comorbidities including ADHD, depression, anxiety, rage attacks, and OCD in patients with TS.

Based on these clinical observations and the fact that the endocannabinoid system (ECS) plays a paramount role in basal ganglia function by modulating the activity of key neurotransmitters including dopamine, glutamate, and GABA and thereby, influences different motor responses [10], alternatively, a “cannabinoid hypothesis” has been suggested in TS [1].

Our study was designed to further explore the involvement of the ECS in the pathophysiology of TS. Therefore, we measured cerebrospinal fluid (CSF) levels of endocannabinoids in a large cohort of adult patients with TS compared with a control group. More specifically, we assessed levels of the two most important endocannabinoids “N”-arachidonoylethanolamine (AEA, anandamide) and 2-arachidonoylglycerol (2-AG) as well as the endocannabinoid-like molecule palmitoyl ethanolamide (PEA) and the lipid arachidonic acid (AA) using liquid-liquid lipid extraction and simultaneous quantification by liquid chromatography multiple reaction monitoring [11].

## Materials and methods

### Subjects

In this prospective study, 20 adult patients with TS according to DSM-5 were included (between 11/2015 and 2/2016). In all patients, the diagnosis of TS was confirmed by one of the authors (KMV). Patients suffering from other severe psychiatric or neurological diseases (such as schizophrenia, alcoholism, epilepsy, mental retardation) and any kind of autoimmune disease were excluded. Patients and controls were asked about use of cannabis and CSF levels of exocannabinoids were measured. In all patients contraindications for lumbar puncture were excluded and a cerebral Magnetic Resonance Imaging (cMRI) was performed (for further details see [12]). All subjects gave written informed consent before entering the study. This study was approved by the local ethics committee (no. 6987) at Hannover Medical School.

In all patients, extensive clinical assessments were performed at the day of lumbar puncture to assess severity of both tics and psychiatric comorbidities by using the following established clinical assessments: Yale Global Tic Severity Scale – Total Tic Score (YGTSS-TTS) [13], Premonitory Urge for Tics Scale (PUTS) [14], Gilles de la Tourette-Syndrome Quality of Life Scale (GTS-QOL) [15], Beck Depression Inventory-II (BDI-II) [16], Conner’s Adult ADHD Rating Scale (CAARS) [17], DSM-IV symptom list for ADHD [18], Wender Utah Rating Scale short version (WURS-K) [19], Beck Anxiety Inventory (BAI) [20], Yale–Brown Obsessive Compulsive Scale (Y-BOCS) [21], and Brief Symptom Inventory (BSI) [22].

In parallel, a control group (*n* = 20, 13 females, mean age = 45 (±20 SD, range, 18–79 years)) was recruited (between 03/2017 and 09/2017) at the Department of Neurology at Hannover Medical School. The control group consisted of patients suffering from either normal pressure hydrocephalus (NPH) or idiopathic intracranial hypertension (IIH) (diagnoses were confirmed by one of the authors (KWS)), who received lumbar puncture for therapeutic reasons. Hence we decided to include only patients with these diagnoses to control disease related CSF changes the control group could not exactly be age or sex matched. None of the controls was treated with cannabis-based medicines or used cannabis recreationally, but six patients with IIH received treatment with acetazolamide.

### Lumbar puncture and routine CSF analyses

Lumbar puncture was performed in all subjects in a sitting position at the same daytime (patients with TS: between 12.00 p.m. and 3.10 p.m., controls: between 10 a.m. and 2 p.m.). In patients with TS, all lumbar punctures were done by one of the authors (KWS). All subjects were in a non-fasting status. All CSF samples were frozen at −80 °C within 20 (TS) and 60 (controls) minutes, respectively, and were not thawed before shipment to Mainz for further analyses. To exclude a significant influence of different freezing times, the effect of different freezing times on endocannabinoid concentrations was assessed in a separate experiment (see supplementary Fig. 1). In all TS patients, in addition, routine parameters were assessed including CSF cell count, cytology, blood-CSF barrier function by CSF-serum albumin quotients (QAlb), and oligoclonal bands (OCB) (for further details see [12]).

### Measurement of endocannabinoid levels

All samples were measured at the Lipidomics/Mass Spectrometry Facility at the Institute for Physiological Chemistry at the University Medical Center Mainz. Endocannabinoids were extracted from 500 µL CSF using a previously reported liquid-liquid extraction [11, 23, 24] which was adapted to the specific CSF volume, i.e., 500 µL. Briefly, frozen CSF was allowed first to thaw on ice (4 °C). 500 µL of ethylacetate/n-hexane (9:1, v/v) containing deuterated internal standards of the endocannabinoids, arachidonic acid and endocannabinoid-like lipids (PEA and oleoylethanolamide (OEA)). The resulting mixture was vortexed for 30 s and centrifuged for 10 min at 20000 g at 4 °C resulting in two-phase separation. The hence resulting mixture was allowed to freeze at −20 °C for 10 min, the upper organic phase was recovered and evaporated to dryness under a stream of nitrogen. The dry lipid extracted was solubilized in 50 µl acetonitrile/water from which 10 µL were injected into liquid chromatography/multiple reaction monitoring (LC/MRM) instrument. The LC/MRM analytical conditions and parameters for qualitative and quantitative analysis were as described elsewhere [11, 23–25]. The endocannabinoid levels determined by LC/MRM were normalized to the CSF volume. Levels of AEA, 2-AG, PEA, and AA—from which both endocannabinoids are derived and/or produced—were measured.

### Measurement of exocannabinoids

Serum levels of THC and its metabolites 11-hydroxy-THC (THC-OH) and 11-nor-delta-9-tetrahydrocannabinol-9-carboxylic acid (THC-COOH) were measured in all subjects (patients and controls) at the Forensic Toxicological Centre Munich to assess levels of exocannabinoids. The procedure used—liquid/liquid extraction followed by LC/MRM—is accredited under DIN EN ISO 17025 with the following limits of detection (LOD) and quantitation (LOQ): 0.05 ng/ml and 0.20 ng/ml for THC, 0.03 ng/ml and 0.12 ng/ml for THC-OH and 0.76 ng/ml and 2.62 ng/ml for THC-COOH, respectively.

### Statistical analyzes

All statistics were calculated using SPSS and Graph Pad Prism 7.03. For statistical analysis a *p* value < 0.05 was considered significant. All statistical analyses were performed using two-tailed testing. As all dependent variables were normally distributed (tested using Kolmogorow–Smirnow test), parametric tests were used throughout. Due to the small sample sizes, we assumed variance homogeneity for all tests. For multiple comparisons Bonferroni correction was used.

### Data availability statement

Any anonymized data not published within this or a related [12] article will be shared by request from any qualified investigator.

## Results

In this study, we included adult patients with TS (*n* = 20, 2 females, mean age = 36.1 (±14.34 SD, range, 19–64 years), mean age at tic onset = 7.7 years (±2.8 SD, range, 3–13 years), and mean tic severity (YGTSS-TTS) = 23.2 (±9.1 SD, range, 10–39)). For further clinical details see Table 1 and [12]. Seventeen patients were unmedicated, one patient received medication for tics with aripiprazole, and two patients reported treatment with cannabis-based medicines: dronabinol (P4: THC 0.94 ng/ml, THC-OH ~0.42 ng/ml (<LOQ), THC-COOH 12 ng/ml) and nabiximols (P9). However in the latter patients no exocannabinoids were detected (N/D). Although none of the patients or the controls reported recreational use of cannabis, THC and/or THC-COOH levels were found to be positive in two other patients (P15: THC N/D, THC-OH N/D, THC-COOH 2.73 ng/ml and P20: THC 0.39 ng/ml, THC-OH N/D, THC-COOH 1.89 ng/ml (<LOQ)).

**Table 1:** Clinical characteristics of patients with TS (for individual data please refer to [12]).

Assessments	*N*	Mean	SD	Range
Age (male, *n* = 18, female, *n* = 2)	20	36.1	14.3	19–64
YGTSS-TTS	20	23.2	9.1	10–39
Y-BOCS	20	8	5.9	0–17
PUTS	20	25.8	5.7	13–36
BDI-II	20	11.8	7.9	0–29
BAI	20	10.9	8.5	0–31
WURS-K	20	23.3	14.7	0–60
CAARS, t-score	17	54.8	12.8	25–90
DSM-IV symptom list for ADHD				
Inattention	20	3.3	2.2	0–8
Hyperactivity/Impulsivity	20	2.7	2.3	0–8
BSI, *t* score	20	61.5	10.7	41–80

*TS* Tourette syndrome, *YGTSS-TTS* Yale Global Tic Severity Scale–Total Tic Score, *Y-BOCS* Yale–Brown Obsessive Compulsive Scale, *PUTS* Premonitory Urge for Tics Scale, *BDI-II* Beck Depression Inventory-II, *BAI* Beck Anxiety Inventory, *WURS-K* Wender Utah Rating Scale short version, *CAARS* Conner’s Adult ADHD Rating Scale, *ADHD* attention-deficit/hyperactivity disorder, *BSI* Brief Symptom Inventory.

Routine cMRI did not reveal any significant abnormalities. Routine CSF analyses demonstrated normal cell count in all patients and only slightly dysfunctional blood-CSF-barrier in 4/20 patients (using QAlb), but positive OCB in CSF only (type 2) in 4/20 patients (P 2, 7, 12, 14) (for further details see [12]).

**Fig. 1:**
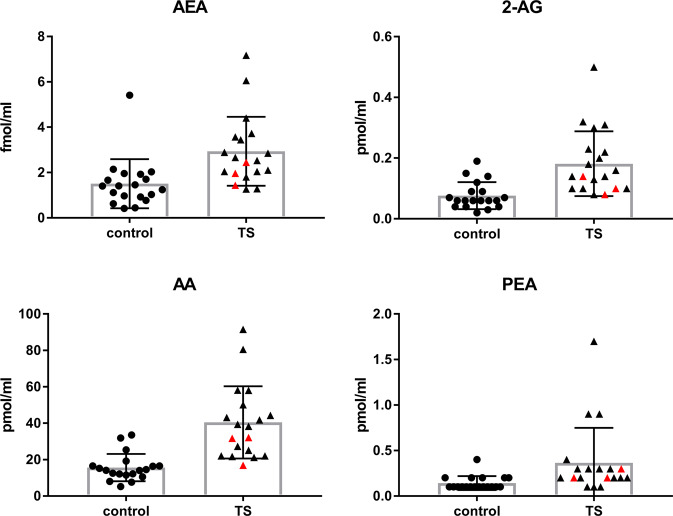
CSF levels of AEA, 2-AG, AA, and PEA in patients with TS compared with controls. All values except AEA (fmol/ml) are given in pmol/ml CSF. AEA “N”-arachidonoylethanolamine, 2-AG 2-arachidonoylglycerol, PEA palmitoyl ethanolamide, AA arachidonic acid, TS Tourette syndrome, CSF cerebrospinal fluid. In red triangles patients who were tested positive for exocannabinoids in serum.

Results of CSF endocannabinoids in one control were classified as outliers (C5), since all measured endocannabinoid concentrations were far outside normal ranges (>mean + 2 SD) (see Table 2). Therefore, for further analyses results of 20 patients with TS and 19 controls (without C5) were used. Levels of AEA, 2-AG, PEA, and AA were significantly elevated in patients with TS compared with controls: AEA (mean ± SD): 2.94 ± 1.52 fmol/ml CSF (TS) vs 1.51 ± 1.08 fmol/ml CSF (controls) vs, *p* = 0.0018; 2-AG: 0.18 ± 0.11 pmol/ml CSF (TS) vs 0.076 ± 0.04 pmol/ml CSF (controls), *p* = 0.0003; PEA: 0.37 ± 0.38 pmol/ml CSF (TS) vs 0.14 ± 0.08 pmol/ml CSF (controls), *p* = 0.02; AA: 40.47 ± 19.84 pmol/ml CSF (TS) vs 15,62 ± 7.49 pmol/ml CSF (controls), *p* < 0.0001 (for individual values see Table 2 and Fig. 1).

**Table 2:** CSF levels of AEA, 2-AG, AA, and PEA in patients with TS (P) compared with controls (C). All values except AEA (fmol/ml) are given in pmol/ml CSF.

Patients no.	AEA	2-AG	AA	PEA	Controls no	diagnosis	AEA	2-AG	AA	PEA
P1	2.10	0.08	25.1	0.2	C1	IIH	1.9535	0.09	31.9	0.1
P2	2.04	0.10	27.3	0.1	C2	IIH	0.4200	0.04	5.2	0.1
P3	1.27	0.20	22.0	0.1	C3	IIH	2.0312	0.04	16.4	0.4
P4	1.96	0.10	16.9	0.2	C4	IIH	0.7710	0.12	14.5	0.1
P5	1.80	0.14	21.7	0.3	C5	NPH	33.0855	6.61	28.9	1.4
P6	3.57	0.30	58.1	0.3	C6	IIH	1.6629	0.06	19.2	0.2
P7	1.28	0.18	21.2	0.3	C7	IIH	1.2457	0.06	15.2	0.2
P8	2.66	0.32	39.4	0.9	C8	IIH	1.7003	0.06	12.5	0.1
P9	3.06	0.10	44.3	0.2	C9	IIH	1.4558	0.03	10.5	0.1
P10	4.40	0.23	38.3	0.9	C10	NPH	2.1462	0.09	25.4	0.2
P11	3.44	0.10	50.1	0.2	C11	NPH	1.9218	0.19	14.1	0.1
P12	2.53	0.10	22.0	0.1	C12	IIH	1.4011	0.15	16.5	0.1
P13	2.85	0.31	44.3	0.2	C13	IIH	0.9667	0.07	16.5	0.2
P14	2.89	0.14	58.0	0.3	C14	IIH	1.0300	0.06	12.1	0.1
P15	2.45	0.14	31.7	0.2	C15	IIH	0.4431	0.07	8.0	0.1
P16	6.06	0.50	91.5	0.2	C16	NPH	1.1134	0.06	12.2	0.1
P17	2.04	0.16	41.8	0.4	C17	IIH	0.9264	0.04	7.6	0.1
P18	3.72	0.13	43.1	0.2	C18	NPH	0.6243	0.02	14.0	0.2
P19	7.17	0.22	80.5	1.7	C19	IIH	5.4088	0.14	33.5	0.1
P20	1.44	0.08	32.1	0.3	C20	IIH	1.4069	0.06	11.5	0.1
mean + SD^a^	2.94 ± 1.52	0.18 ± 0.11	40.47 ± 19.84	0.37 ± 0.38			1.51 ± 1.08	0.076 ± 0.04	15.62 ± 7.49	0.14 ± 0.08
*p* value							0.0018	0.0003	<0.0001	0.02

*AEA* “N”-arachidonoylethanolamine, *2-AG* 2-arachidonoylglycerol, *PEA* palmitoyl ethanolamide, *AA* arachidonic acid, *CSF* cerebrospinal fluid, *P* patient with TS, *C* control, *IIH* idiopathic intracranial hypertension, *NPH* normal pressure hydrocephalus.

^a^For controls not included outlier C5; *p* value: patients with TS (*n* = 20) compared with controls (*n* = 19).

When comparing CSF values of endocannabinoids with clinical data in patients with TS, we found no influence of patients’ sex, age, and medication (including cannabis-based medication). Neither tic severity (according to YGTSS-TTS) and age of tic onset, nor severity of OCD (according to Y-BOCS) and depression (according to BDI-II) correlated with CSF levels of AEA, 2-AG, and AA. However, after multiple test correction we found a significant correlation between the severity of ADHD (according to WURS-k) and CSF levels of 2-AG (p < 0.01). For the correlation between the severity of ADHD (according to CAARS and WURS-k) and CSF concentrations of AA a trend was observed (for further details see Table 3).

**Table 3:** Correlation of CSF endocannabinoids with clinical data in patients with TS.

	Age	Age at tic onset	Tic severity (YGTSS-TTS)	OCD (YBOCS)	Premonitory urges (PUTS)	ADHD (CAARS)	ADHD (WURS-K)	Depression (BDI-II)
AEA								
Pearson correlation	0.179	0.020	−0.122	0.014	0.131	−0.337	0.365	0.199
Sig. (2-tailed)	0.449	0.935	0.607	0.953	0.583	0.185	0.113	0.400
N	20	20	20	20	20	17	20	20
2-AG								
Pearson correlation	−0.121	0.108	−0.036	0.111	0.097	0.171	0.**750**^a^	0.241
Sig. (2-tailed)	0.611	0.649	0.881	0.641	0.685	0.512	0.000	0.307
N	20	20	20	20	20	17	20	20
AA								
Pearson correlation	0.147	0.022	−0.019	0.137	0.001	−0.637	0.593	0.023
Sig. (2-tailed)	0.536	0.925	0.936	0.566	0.996	0.006	0.006	0.924
N	20	20	20	20	20	17	20	20
PEA								
Pearson correlation	−0.003	0.053	−0.233	−0.193	0.467^a^	−0.113	0.186	0.337
Sig. (2-tailed)	0.989	0.824	0.322	0.414	0.038	0.667	0.433	0.146
N	20	20	20	20	20	17	20	20

*TS* Tourette syndrome, *CSF* cerebrospinal fluid, *AEA* “N”-arachidonoylethanolamine, *2-AG* 2-arachidonoylglycerol, *PEA* palmitoyl ethanolamide, *AA* arachidonic acid, *YGTSS-TTS* Yale Global Tic Severity Scale–Total Tic Score, *OCD* obsessive-compulsive disorder, *Y-BOCS* Yale–Brown Obsessive Compulsive Scale, *PUTS* Premonitory Urge for Tics Scale, *ADHD* attention-deficit/hyperactivity disorder, *CAARS* Conner’s Adult ADHD Rating Scale, *WURS-K* Wender Utah Rating Scale short version, *BDI-II* Beck Depression Inventory-II.

^a^Significant after Bonferroni correction for multiple comparisons.

There was no association between CSF levels of AEA, 2-AG, PEA, and AA and abnormal routine CSF findings (dysfunctional blood-CSF-barrier, positive OCB type 2). Medication with nabiximols and dronabinol had no influence on endocannabinoid levels. Even after exclusion of those two patients, who had received treatment with cannabis-based medicines, and those two additionally tested positive, all results remained statistically significant with a slight increase in the difference between means among the groups. In those patients with positive THC/THC-COOH levels, levels of AEA, 2-AG, PEA, and AA were at the lower end of the range (see Fig. 1).

## Discussion

This is the first study demonstrating alterations in endocannabinoid levels in adult patients with TS. We found significant elevations of both the endocannabinoids AEA and 2-AG, the endocannabinoid-like ligand PEA, and the metabolite AA in adult patients with TS compared with controls. CSF levels of 2-AG correlated with severity of ADHD. As a trend ADHD symptoms were further correlated to CSF levels of AA. No other correlations could be detected, neither with further clinical data, nor with routine CSF abnormalities.

Alterations of CSF endocannabinoid levels in TS can be interpreted in different ways. First, it can be speculated that elevations of AEA, 2-AG, PEA, and AA are secondary in order to compensate for the presumed striatal dopaminergic hyperinnervation underlying TS. The striatum contains high levels of central cannabinoid CB1 receptors [26]. Although nigrostriatal dopaminergic neurons appear not to contain CB1 receptors, the ECS significantly influences the activity of the dopaminergic system, resulting in clinically relevant alterations of motor activity [10, 27]. In addition to this indirect effect, it has been shown that striatal dopaminergic transmission is also modulated directly via vanilloid TRPV1 receptors [28] a receptor functionally related to the cannabinoid signaling system—and cannabinoid CB2 receptors located on dopaminergic neurons [27]. On the other hand, stimulation of dopamine D2-like receptors increases the levels of AEA in the striatum [29]. Endocannabinoids may counteract the effects of dopamine D2 receptor stimulation, since dopamine D2 receptor-dependent stimulation of the ECS results in an inhibitory feedback mechanism [29]. Thus, in the striatum, there is not only a complex indirect functional interaction between CB1 and dopamine receptors, but endocannabinoids also control directly the dopaminergic neurotransmission [10].

Assuming both that TS is caused by a dopaminergic hyperinnervation and that increased levels of endocannabinoids represent a compensatory mechanism, one would expect that endocannabinoids may reduce increased phasic or tonic dopamine or both. In fact, several lines of evidence support the hypothesis that endocannabinoids may reduce striatal dopaminergic signaling: (i) AEA is a full agonist for TRPV1 receptors [30] and activation of TRPV1 receptors located in nigrostriatal dopaminergic neurons results in direct inhibition of dopamine activity [31]. Therefore, AEA has been referred to a “stop signal for dopamine” [29], (ii) AEA behaves as a hypokinetic substance [31]; (iii) activation of TRPV1 receptors produces hypokinesia in rats [32]; (iv) AEA induced hypokinesia is mediated by a reduction in depolarization-induced neurotransmitter release on dopaminergic terminals [31]; (v) dopamine transmission can be modulated via G protein/adenylyl cyclase signal transduction mechanisms shared by both CB1 and dopamine D1/D2 receptors located in basal ganglia neurons [10]; and (vi) only recently, also cannabinoid CB2 receptors have been identified on nigrostriatal dopaminergic neurons [33] suggesting that direct activation of these receptors also modulates striatal dopaminergic transmission [27].

In summary, it can be speculated that in TS dopaminergic hyperinnervation leads to a compensatory increase in endocannabinoids in order to decrease dopaminergic transmission. This assumption is also in line with reported beneficial treatment effects of cannabis-based medicines in TS. In addition, in a pilot study in adult patients with TS (*n* = 20), it could be demonstrated that further increase of 2-AG by ABX-1431, a highly selective inhibitor of the degradation enzyme monoacylglycerol lipase (MAGL), results in a reduction of tics and premonitory urges [34].

Secondly, it can be speculated that increased endocannabinoids in TS are related to alterations in other neurotransmitter systems than the dopaminergic system, since CB1 receptors are also present on striatal GABAergic interneurons, cholinergic interneurons as well as glutamatergic neurons (for review see [10]). All these neurotransmitters have also been suggested to play a role in the pathophysiology of TS [35].

Thirdly, one might also speculate that alterations in the ESC – and not in other neurotransmitter systems such as the dopaminergic system – represent the primary cause of TS. However, until today there are no other genetic [36], imaging [37], or biochemical studies available reporting about alterations in the ECS in patients with TS.

Fourthly, from our results it cannot be excluded that elevated endocannabinoid levels are simply an epiphenomenon with no pathological relevance.

In humans, a physiological “endocannabinoid tone” has been suggested based on tonically released endocannabinoids [38] and an intact interplay between the endocannabinoids including their production and metabolism and cannabinoid CB1/CB2 and other cannabinoid-like receptors (such as TRPV1 receptors) [39]. Alterations of endocannabinoid levels have been described in several different neurological disorders including Parkinson’s disease [40], epilepsy [41], and Multiple Sclerosis [42]. Migraine and post-traumatic stress disorder (PTSD) have been suggested to represent so called “endocannabinoid deficiency syndromes”, since in chronic migraine reduced CSF levels of AEA have been detected [43], while patients with PTSD demonstrated an up-regulation of CB1 receptors [44].

Based on clinical findings demonstrating beneficial effects of cannabis-based medicine in patients with TS, one might speculate that TS is also caused by an “endocannabinoid deficiency”. Our results of *elevated* endocannabinoid levels do not contradict with this hypothesis, since it can be speculated that the overall endocannabinoid tone is still reduced in TS, for example due to a reduced number or reduced sensitivity of cannabinoid CB1/CB2 receptors or an overactivity of the degradation enzymes MAGL (for 2-AG) and fatty acid amide hydrolysis (FAAH, for AEA). Such an overall deficient endocannabinoid tone might result not only in a compensatory increase of endocannabinoid levels, but also dysfunctions in several other neurotransmitter system including the dopaminergic, GABAergic, and glutamatergic systems [10]. This hypothesis in turn would provide a plausible explanation for the fact that several different neurotransmitter systems have been suggested to be involved in the pathophysiology of TS including the dopaminergic, GABAergic, glutamatergic and other systems [35].

Furthermore, it is believed that the ECS—in particular a dysregulation of 2-AG signaling—plays an important role in the regulation of chronic stress [45]. Stress, in turn, is the most relevant environmental factor that increases tics [46]. In this context, it is remarkable that stress modulates the ECS by altering the expression level of CB1 receptors, increases 2-AG and reduces AEA (for review see [47]). Since stress-induced reduction of AEA occurs rapidly [47], it can be speculated that deterioration of tics by stress is caused by decreased AEA levels, which in turn may lead to increased dopaminergic signaling.

When comparing endocannabinoid levels with clinical data, we found correlations between the severity of ADHD and levels of 2-AG. This correlation between endocannabinoid levels and the severity of ADHD is noteworthy, since there is some evidence that the cannabis extract nabiximols is effective in the treatment of ADHD [48] and the activity of FAAH has been found to be decreased in patients with ADHD suggesting dysfunctional AEA degradation in this group of patients [49].

Two patients (P4, P9) received treatment for TS with cannabis-based medicine, however, the latter was tested negative for exocannabinoids. Two further patients (P15, P20) were tested positive for THC or THC-COOH presumably due to recreational use of cannabis. Interestingly, levels of endocannabinoids were at the lower end, but not outside the range found in other patients. In four patients (P 2, 7, 12, 14), we found positive OCBs (further results were reported elsewhere [12]). This result clearly indicates a pathological immune process in terms of an intrathecal production of IgG antibodies. However, we found no differences in endocannabinoid levels with respect to positive OCBs suggesting that elevated endocannabinoid levels in TS are unrelated to these immunological changes.

The following limitations of the study have to be addressed: (i) the sample size is still relatively small. However, this is the first study investigating endocannabinoid CSF levels in TS and lumbar puncture is not routinely performed in patients with TS; (ii) typically tics wax and wane and the assessment of tics (and all other clinical symptoms) were performed only once at the day of lumbar puncture. Since the YGTSS-TTS measures tic severity during the last week, we believe that current tic severity is reflected appropriately; (iii) although patients received a compensation fee for participating in the study, we do not believe that this has caused any bias in patient selection; (iv) our control group consists of patients with IIH and NPH. Due to ethical reasons we did not recruit a group of healthy controls. However, including patients with IIH and NPH as controls is common practice in CSF studies [50] and there is no evidence suggesting alterations in the ECS in these pathologies; (v) our control group consists of more females and is slightly older compared with the group of patients with TS. While sexual dimorphism in response to cannabinoid compounds has been reported [51], only scarce data are available on gender differences in brain regions, while no evidence on gender differences in circulating endocannabinoid levels have been reported due to our knowledge [52]. Moreover, there is no reported evidence that levels of endocannabinoids in CSF are influenced by sex or this minor age difference [52]; and (vi) data obtained from one control subject (C5) were excluded from further analyses, since results of endocannabinoids were far outside the normal ranges at our lab. Even a critical review (including clinical data, procedure of lumbar puncture, further processing with CSF in the labs at Hannover Medical School and the Institute of Physiological Chemistry Mainz) did not lead to a conclusive explanation for this finding. However, even when not excluding this outlier from further analyses, CSF levels of AEA were still significantly elevated.

In conclusion, we were able to demonstrate for the first time elevated CSF levels of AEA, 2-AG, PEA, and AA in patients with TS suggesting that the ESC is involved in the pathophysiology of the disease. However, it cannot entirely be excluded that elevated endocannabinoid levels are simply an epiphenomenon. Because of the complex bidirectional interaction between the ECS and several other neurotransmitter systems, one might speculate that increased levels of endocannabinoids represent a secondary mechanism in order to compensate for dopaminergic hyperinnervation or, alternatively, represent a primary cause of TS and changes in other neurotransmitter systems are secondary due to the complex interaction between these systems.

## Funding and disclosures

This work was partly supported by the Else Kröner-Fresenius-Stiftung within the KlinStrukMed programme 2015-2016 of the Hannover Biomedical Research School. KRMV has received financial or material research support from the EU (FP7-HEALTH-2011 No. 278367, FP7-PEOPLE-2012-ITN No. 316978), the German Research Foundation (DFG: GZ MU 1527/3-1), the German Ministry of Education and Research (BMBF: 01KG1421), the National Institute of Mental Health (NIMH), Tourette Gesellschaft Deutschland e.V., Else-Kröner-Fresenius-Stiftung, and GW, Almirall, Abide Therapeutics, Lundbeck, Syneos Health, and Therapix Biosiences, consultant's honoraria from Abide Therapeutics, Tilray, Resalo Vertrieb GmbH, Columbia Care, Bionorica Ethics GmbH, Eurox Deutschland GmbH, and Therapix Biosiences, royalties from Medizinisch Wissenschaftliche Verlagsgesellschaft Berlin, Elsevier, and Kohlhammer, and speaker’s fees from Tilray, Emalex, Cogitando GmbH, and Wayland group. She holds shares of Nomovo Pharm. TS received honoraria from Alexion, Bayer Vital GmbH, CSL Behring, Merck, Novartis, Sanofi-Aventis, all outside the submitted work. LB reports no disclosures. BL reports no disclosures. FM reports no disclosures. CB reports no disclosures. KWS reports no disclosures. The authors declare that there are no competing financial interests in relation to the work described. Open access funding provided by Projekt DEAL.

## Supplementary information

Supplemental Figure 1:

Supplemental figure legend
