# Supplementary figures and images for: Cerebrospinal fluid endocannabinoid levels in Gilles de la Tourette syndrome
Source: Neuropsychopharmacology. 2020 Apr 9;45(8):1323–9. doi: 10.1038/s41386-020-0671-6 (PMC7297729; doi:10.1038/s41386-020-0671-6)

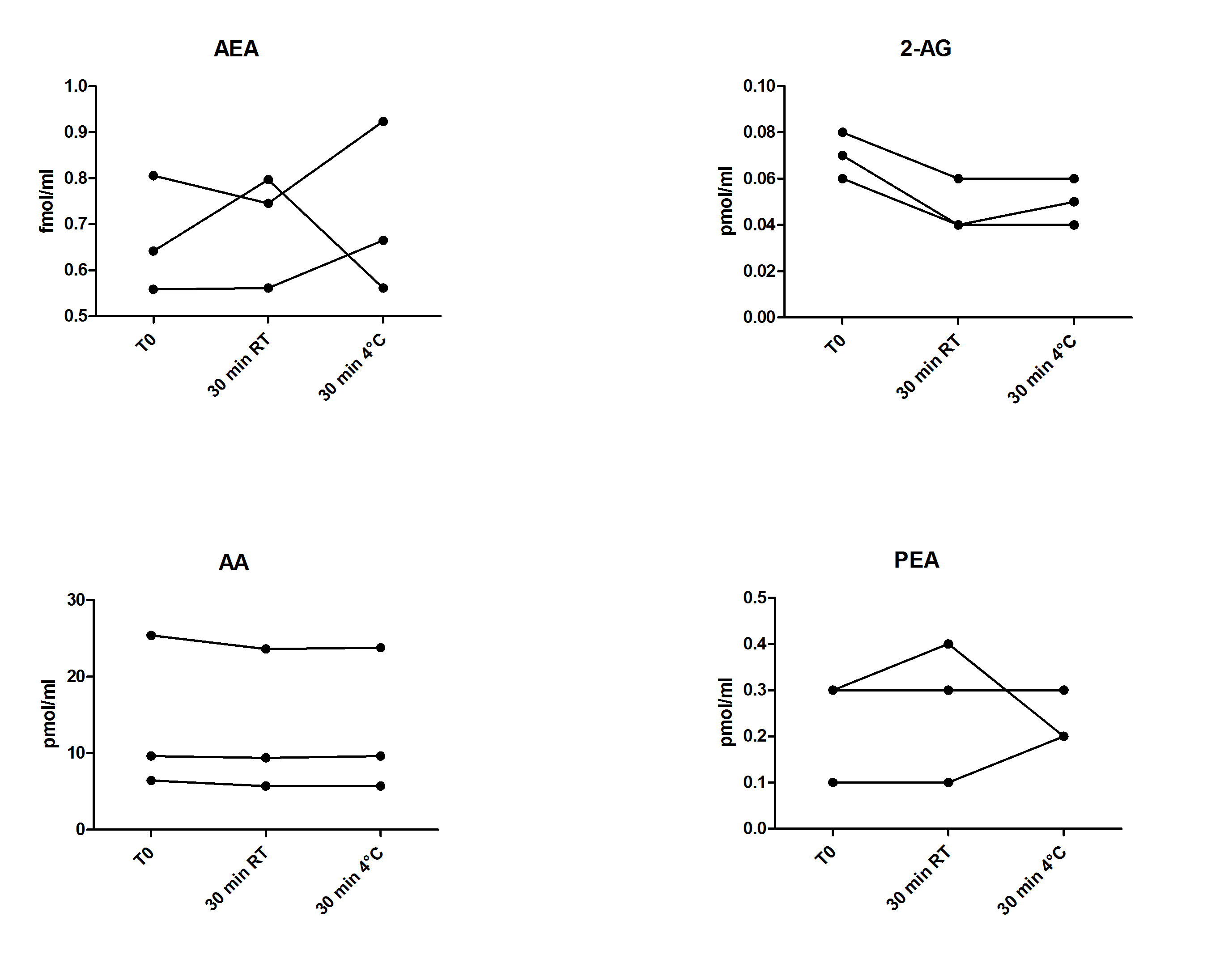

Supplement: Supplementary file 1 — Supplemental Figure 1: [file 41386_2020_671_MOESM1_ESM.jpg]
