# Supplementary material for: Cerebrospinal fluid endocannabinoid levels in Gilles de la Tourette syndrome
Source: Neuropsychopharmacology. 2020 Apr 9;45(8):1323–9. doi: 10.1038/s41386-020-0671-6 (PMC7297729; doi:10.1038/s41386-020-0671-6)
Supplement: Supplementary file 2 — Supplemental figure legend [file 41386_2020_671_MOESM2_ESM.docx]

**Supplemental Figure 1:**

Cerebrospinal fluid (CSF) from three patients was split in three aliquots for stability testing: one aliquot was immediately frozen (T0) one was kept at room temperature for 30 min (T1) and one maintained at 4 °C (T2) before freezing (- 80 °C). As can be seen, a significant influence of different freezing times on endocannabinoid concentrations can be excluded.
